# The Examination of the Urine for Sugar

**Published:** 1908-02-08

**Authors:** 


### Pathology in General Practice.

### THE EXAMINATION OF THE URINE FOR SUGAR.

The examination of urine for sugar is, as a rule, a very simple proceeding. Fehling's test is generally employed as a matter of routine, and this enables one, in most cases, to pronounce quite decidedly for or against the presence of sugar in any sample of urine. But there remain a few cases, such as every practitioner must have met with from time to time, when a green or black precipitate, or it may be only a discoloration, appears on the addition of Fehling's reagent, suggesting that the urine is not quite normal, although one hesitates to condemn it as containing sugar. These cases are extremely puzzling, and very often lead to an incorrect diagnosis; or else the suspicious urine is sent to a clinical laboratory for further examination. In this and a succeeding article it is proposed to describe briefly the general characteristics of urine containing sugar, to give an outline of the commoner qualitative tests, with a reference to the value and the fallacies of each, and to call attention to the safranin test, which, on account of its ease and reliability, deserves wider recognition than it has yet received.

With regard to the occurrence of sugar in the urine of healthy persons, the present opinion is that normal urine often, if not always, contains a trace of sugar—usually less than ·1 per cent.—which can be detected by the safranin test, but not by Fehling's reaction. Any quantity greater than ·2 per cent. is to be regarded as pathological. In diabetes the urine is of high specific gravity (1030 to 1040), the amount passed in twenty-four hours is increased, often to 10 pints or more, and in consequence of this dilution the colour is pale. The acidity is often accentuated, so that there may be no alkaline tide after meals or after the administration of alkaline drugs. Albuminuria may occur in chronic diabetes, and almost always accompanies the onset of coma. The amount of sugar excreted varies greatly according to the severity of the disease. In an average case 3 litres (100 oz.) of urine may be passed in twenty-four hours, containing 100 grammes of sugar. This is equivalent to 3 to 4 per cent. of sugar, or about 15 grains to the ounce. As a rule, the urine passed after a meal contains a large proportion of sugar, whereas there is but little in the early-morning sample. For this reason it is most important that a twenty-four hours' specimen should be taken for analysis. The sugar present in both normal and diabetic urine seems to consist almost entirely of dextrose. After parturition, lactose may be found in the urine of healthy patients, whilst maltose and laevulose are occasionally excreted in diabetes; but these substances are present in such small amounts that the total sugar may be returned as dextrose without fear of serious error.

A very large number of tests for sugar in urine have been devised, but of these only three are in common use, namely, Fehling's test, the phenyl-hydrazine test, and the fermentation test.

*Fehling's Test.*—This is one of a large group of tests depending on the powerful reducing-action of dextrose on various metallic compounds. This reduction is facilitated by a high temperature and the presence of an alkali, and under these conditions dextrose will reduce the oxides of silver, bismuth, and mercury to the metallic state, cupric to cuprous salts, and ferricyanides to ferrocyanides. The metal employed in Fehling's reaction is, of course, copper, and the rationale of the proceeding is as follows:—If an alkali be added to a solution of a salt of copper, a pale-blue precipitate of hydrated cupric oxide is formed. This precipitation, which would interfere with any test for reduction, can be inhibited by the addition of such bodies as tartaric acid, citric acid, or glycerin, which render the cupric oxide soluble. If, then, a mixture be made of the copper salt, the alkali, and the inhibiting agent, no precipitation occurs; but on boiling this solution with a reducing agent, such as dextrose, the insoluble cuprous hydroxide appears as a yellow precipitate, which, on further heating, loses a molecule of water, and is converted into the orange-red cuprous oxide. Tartaric acid is the inhibiting substance in Fehling's reagent, which is a solution of double tartrate of copper (cupric) and sodium or potassium, with a considerable amount of caustic alkali. The reagent is made up in two parts in the following manner:—The copper solution is prepared by dissolving 35 grammes of pure copper sulphate in 500 c.c. of distilled water, and filtering if necessary. The alkaline solution is prepared by dissolving 180 grammes of Rochelle salt (potassium sodium tartrate) in 300 c.c. of hot water, filtering, and adding 100 grammes of caustic potash (or 70 grammes of caustic soda); when cool, the mixture is made up to 500 c.c. with distilled water. It is most important that these two solutions should be kept in separate bottles until actually required for use.

The best way of applying Fehling's test is to take in one test-tube 2 c.c. of both the copper and the alkaline solutions; in a second test-tube take 4 c.c. of the urine. Bring the contents of these two tubes to the boiling-point simultaneously, and then remove from the flame. After 10 to 15 seconds, when the temperature will have fallen some degrees below the boiling-point, pour the urine into the tube containing the Fehling's reagent, and watch for any reduction. In some cases, an orange precipitate of cuprous oxide will appear almost immediately. In others the reaction will require a minute or two to develop: thus, ·4 per cent. of sugar will generally give a precipitate in one minute, whereas ·25 per cent. may not do so in less than two minutes. If the specific gravity of the urine is very high (1040), it is advisable to lower it to 1015, or thereabouts, by the addition of water, before applying the test.

(*To be concluded.*)

